# PP84 From Regulatory Approval To Public Health System Coverage: Overcoming Challenges For Timely Access To Oncology Drugs In Brazil

**DOI:** 10.1017/S0266462325103012

**Published:** 2025-12-29

**Authors:** Stéfani Borges, Mateus Cerqueira, Gustavo Frio, Bruna Santos, Ivan Zimmermann

## Abstract

**Introduction:**

Access to oncology drugs in Brazil is influenced by regulatory and economic factors that affect approval and coverage by the public health system. This study examined the time gap between regulatory approval and coverage, considering factors such as request origin and therapeutic class, to understand how to improve access to oncology treatments in Brazil in alignment with global healthcare needs.

**Methods:**

This descriptive quantitative study analyzed data from the Brazilian National Committee for Health Technology Incorporation (CONITEC) recommendation reports for oncology drugs approved for coverage between 2019 and 2023. Key data points, such as the dates of regulatory approval, coverage, therapeutic class, request origin, and drug prices, were collected. These elements were used to map the average time between regulatory approval and integration into the public health system, while also examining their potential impact on incremental cost-effectiveness ratios (ICERs), described in terms of price per quality-adjusted life year (QALY).

**Results:**

Among the 14 oncology drugs covered by the public health system, the time from regulatory approval to CONITEC decision ranged from 2.7 to 25.1 years. Monoclonal antibodies accounted for the largest proportion (43%), followed by protein kinase inhibitors (29%), and proteasome inhibitors (14%). ICERs varied widely, with breast cancer treatments like abemaciclib (BRL649,999.59 [USD108,890.45]/QALY) and palbociclib (BRL437,124.32 [USD 73,157.91]/QALY) showing the highest values. Rituximab (BRL28,564.07 [USD 4,781.06]/QALY) and bortezomib (BRL20,150.59 [USD 3,374.53]/QALY) were below the cost-effectiveness threshold, while alphaepoetin showed a negative ICER as it was more effective and less costly than transfusion support.

**Conclusions:**

This study highlighted the variability in the time it takes for oncology drugs to achieve coverage within the public health system after approval. Key factors like therapeutic class and request origin significantly impact this timeline. Streamlining the coverage process could improve access to critical oncology treatments, enhancing patient outcomes and aligning with global efforts to accelerate medical innovations in healthcare systems.

